# Pleomorphic Liposarcoma of Femur: A Rare Soft Tissue Sarcoma Metastasized to the Bone—Case Report and Review of Literature

**DOI:** 10.1155/2022/9195529

**Published:** 2022-03-07

**Authors:** Thanate Poosiripinyo, Thanapon Chobpenthai, Taweechok Wisanuyotin, Winai Sirichativapee

**Affiliations:** ^1^Khon Kaen Hospital, Department of Orthopaedics, Khon Kaen, Thailand; ^2^Chulabhorn Royal Academy, Princess Srisavangavadhana College of Medicine, Bangkok, Thailand; ^3^Department of Orthopaedics, Faculty of Medicine, Khon Kaen University, Khon Kaen, Thailand

## Abstract

**Background:**

Pleomorphic liposarcoma (PLPS) accounts for less than 5% of liposarcoma, and its metastasis to bone is rare. As a high-grade tumor, PLPS is reported to be more invasive with high local recurrence and distant metastasis. Here, we report a case of PLPS of the femur and undertake a review of the literature. *Case Presentation*. A 58-year-old man presented with a big mass at posterior aspect of his left thigh. The computed tomography of the chest for staging revealed two nodules at the left upper lung field. Wide resection of the soft tissue mass at the left thigh was performed by a general surgeon. Thoracotomy and wedge resection of the lung nodules was carried out by a cardiothoracic surgeon. Pathologic diagnosis suggested PLPS. Three years later, he was presented with sudden right hip pain after he slipped. The plain radiograph revealed an osteolytic lesion at the right proximal femur with minimally displaced pathological fracture. The MRI showed the presence of a tumor at the proximal part of the right femur and its soft tissue invasion. The patient underwent en bloc proximal femur wide resection followed by cemented long-stem bipolar hemiarthroplasty. The final histopathology report from definite surgery specimen revealed pleomorphic spindle, round, and polygonal cells arranged in sheets, short fascicles, and storiform arrays. There were no complications, adverse outcomes, or recurrence reported at six months after surgery. The patient could walk without gait aid and had good functional outcomes according to the TESS questionnaire.

**Conclusion:**

PLPS is a highly aggressive tumor with a high distant metastatic rate. The definite diagnosis of PLPS is made on the basis of histopathology. Surgical treatment involving wide resection that aims to achieve a negative margin is the best option currently available, and we recommend treating bone metastasis from PLPS as the primary site of the tumor. The effect of chemotherapy and radiotherapy in preventing postoperative recurrence is still unclear and requires further studies.

## 1. Introduction

Soft tissue sarcoma (STS) constitutes around 20% of all the adult sarcomas, and liposarcoma is its common subtype [1]. According to the 4th AFIP fascicle, by definition, liposarcoma is a malignant tumor that has differentiation towards adipocytes [2]. Based on adipocyte differentiation, World Health Organization classified liposarcoma into three groups and five types: well-differentiated/dedifferentiated liposarcoma (WDL/DDL), myxoid/round-cell liposarcoma (MRC), and pleomorphic liposarcoma (PLPS) [3]. PLPS accounts for less than 5% of liposarcoma, and its metastasis to bone is rare [4]. As a high-grade tumor, PLPS is reported to be more invasive with high local recurrence and distant metastasis in 30–50% of cases [5, 6]. The commonest site for this tumor is the lower extremity, and less common anatomical sites are the retroperitoneum, lungs, mediastinum, and bones [5, 7]. According to the literature reviews, there are few bone metastasis reports from PLPS [8, 9], and the treatment strategies are still unclear. Although surgical resection is the mainstay of treatment, there is substantial uncertainty about histological diagnosis and the best suitable treatment of this rare tumor [10].

n this case report, we provided an overview of the clinical, radiological, and pathological findings in a case of metastatic PLPS in the proximal femur of a 58-year-old male. We also reviewed the literature about PLPS of bone and proposed revised diagnostic criteria as well as treatment options according to our findings.

## 2. Case Presentation

**Figure 1:**
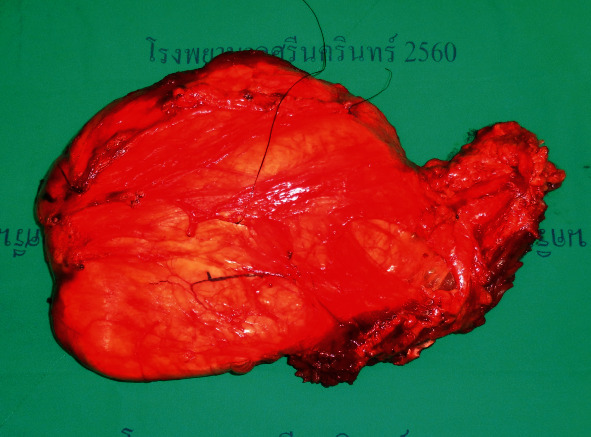
Specimen of soft tissue tumor (the primary tumor) after wide resection.

A 58-year-old man presented with a big mass at posterior aspect of his left thigh. The computed tomography (CT) of the chest for staging revealed two nodules at the left upper lung field. Wide resection of the soft tissue mass at the left thigh was carried out by a general surgeon (Figure 1). Cardiothoracic surgeon performed thoracotomy and wedge resection of the lung nodules. The pathological report of mass at left thigh suggested PLPS, and tumor breakthrough capsule and lung nodules were metastatic PLPS. The patient was treated postoperatively with radiotherapy at left thigh for local control and chemotherapy with 6 cycles of doxorubicin and ifosfamide regimen for systemic control.

**Figure 2:**
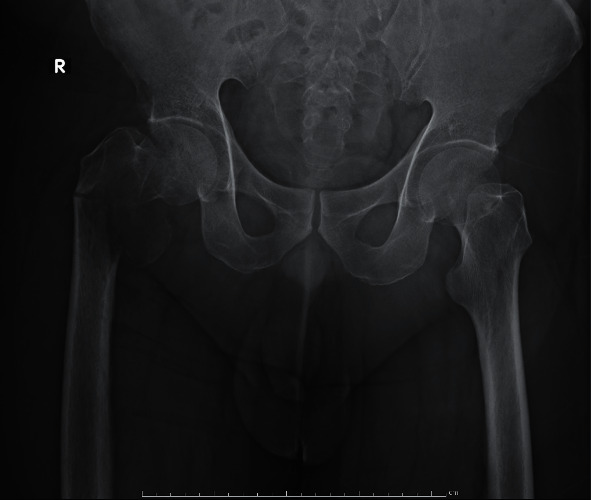
Plain radiograph revealed osteolytic lesion at right proximal femur with minimally displaced pathological fracture at the intertrochanteric region.

**Figure 3:**
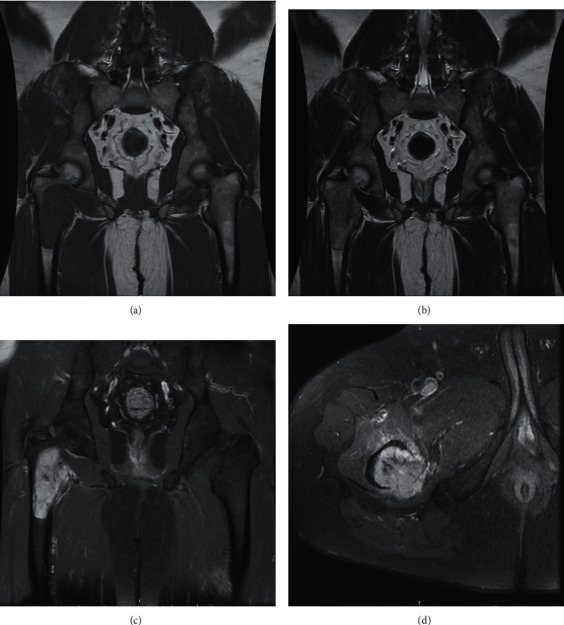
The MRI revealed the tumor at right proximal femur with soft tissue extension, especially at posteromedial aspect: MRI coronal T1 weight (a), coronal T2 weight (b), coronal T1 + contrast (c), and axial T1 + contrast (d).

**Figure 4:**
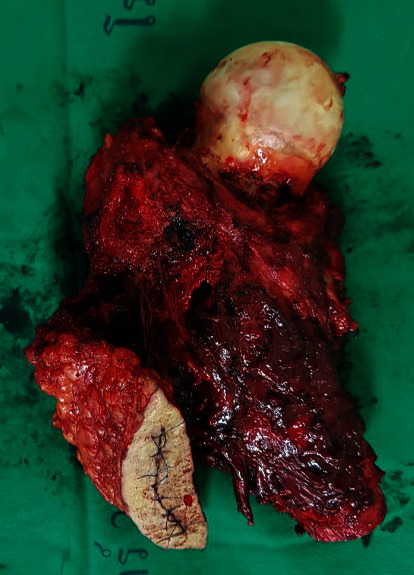
Specimen of proximal femur includes biopsy tract after en bloc resection.

**Figure 5:**
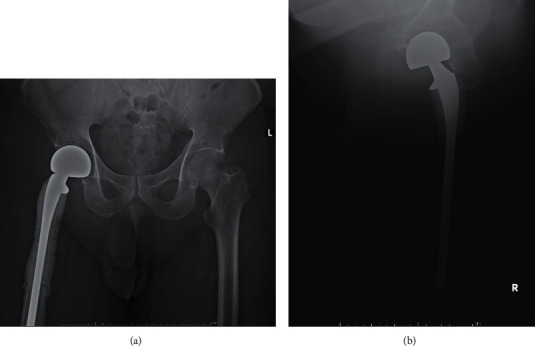
Postoperative radiographs after en bloc resection and reconstruction of the proximal femur: anteroposterior view (a) and trans inguinal view (b).

Three years later, he presented with sudden right hip pain after he slipped and was brought to the hospital by an ambulance. The physical examination revealed that he could not bear weight on his right hip. There was no apparent deformity and palpated mass, and neurovascular was also intact. However, there was tenderness at greater trochanter and limited range of motion due to pain. All of his laboratory investigations were normal. The plain radiograph revealed an osteolytic lesion at the right proximal femur with a minimally displaced pathological fracture at the intertrochanteric region (Figure 2). The MRI showed the presence of tumors at proximal part of the right femur and soft tissue invasion, especially at the posteromedial aspect (Figures 3(a)–3(c)). Femoral neurovascular structures and sciatic nerves were spared from the tumor (Figure 3(d)). The chest CT revealed right upper lung mass with no other skeletal metastasis shown by bone scan. The right upper lung mass was lung metastasis, which was confirmed by a pathologist from core needle biopsy of the lung. The thoracotomy and right upper lobectomy were taken. The patient underwent incisional biopsy through a direct lateral approach. The histopathology report suggested metastatic PLPS. The patient underwent en bloc proximal femur wide resection (Figure 4). Owing to financial problems, the patient was unable to copay for endoprosthesis, and the allograft was not available at that time. The surgeon considered to opt reconstruction by cemented long-stem bipolar hemiarthroplasty (ECHELON revision hip stem, Smith and Nephew Company). Polymethyl methacrylate (PMMA) was used for implant fixation at the proximal femur. In order to restore hip abduction, soft tissue reconstruction was performed, specifically for gluteus medius muscle, using Dacron vascular graft covering PMMA.  Figure 5 shows the postoperative radiographs after the en bloc resection and reconstruction of the proximal femur.

**Figure 6:**
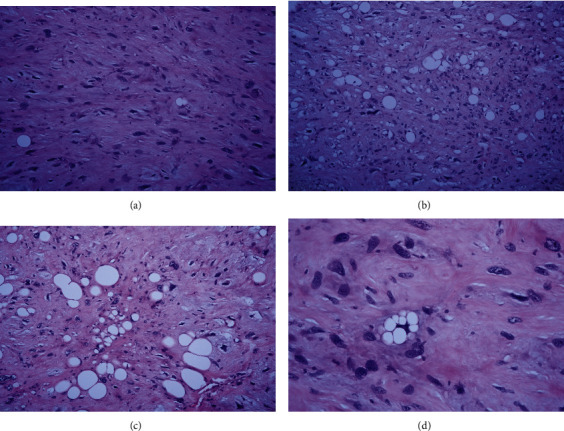
Pathological diagnosis of tissue from right proximal femur: metastatic pleomorphic liposarcoma, with the presence of vascular invasion and all surgical resected margins appear free of tumor. 20× (a, b), 40× (c), and 60× (d).

The final histopathology report from definite surgery specimen revealed pleomorphic spindle, round, and polygonal cells arranged in sheets, short fascicles, and storiform arrays. Severe nuclear atypia with multinucleation and numerous mitoses, including atypical forms of intracellular and extracellular eosinophilic droplets, was presented. Variable size of pleomorphic adipocytes and lipoblasts was identified with vascular invasion. All surgical margins were free from the tumor cell (Figures 6(a)–6(d)). The patient received adjuvant radiotherapy and chemotherapy after the surgery, as per tumor board decision.

The patient was advised for early ambulation and full weight-bearing after the right bipolar hemiarthroplasty. The postoperative course was uneventful, and the patient was discharged within one week of surgery. There were no complications, adverse outcomes, or recurrence reported at 6 months after surgery, and he could walk without gait aid. On the Thai version of the Toronto Extremity Salvage Score (TESS) for patients with bone and soft-tissue sarcoma, he had good functional outcomes with a score of 74.1% [11].

## 3. Discussion

PLPS is a rare soft tissue sarcoma. Bone is the second common site of metastasis of pleomorphic liposarcoma [12]. On the contrary, most skeletal metastases were prostate and breast cancer (up to 70%) [13]. PLPS is the rarest type of liposarcoma [14], accounting for only 5-10% of all liposarcoma subtypes, but is considered as a high-grade malignancy with a high rate of metastasis, local recurrence, and poor prognosis [4–6]. The highest prevalence of PLPS is found in the 6^th^ and 7^th^ decade of life, and it affects both sexes equally [7, 15]. However, some previous studies have also reported cases of younger patients, with the youngest reported case was 8 years of age [16].  Table 1 summarizes the clinical and demographic features of cases of PLPS reported in the literature. In this report, we presented a known case of PLPS of the femur in a 58-year-old male.

In PLPS, there is a rapid growth of a painless mass, which is easily neglected until the mass is large enough or produce symptoms due to compression of surrounding structures [17]. Three distinct morphological features of PLPS have been described in the literature, such as malignant fibrous histiocytoma-like liposarcoma, epithelioid structure like neoplasia, and fusiform/spindle-shaped cell neoplasia [16]. PLPS most commonly arise in the deep soft tissues of the proximal part of the lower extremity, with thigh as the commonest site reported in 34% of the cases; however, it could also occur in the retroperitoneum, lungs, mediastinum, and bones [12, 17]. In our case, the patient presented with a big mass at the posterior aspect of his left thigh in the first episode. After recurrence, he presented with sudden right hip pain after he slipped and was brought to the hospital by an ambulance. The final histopathology report from definite surgery specimen revealed pleomorphic spindle, round, and polygonal cells arranged in sheets, short fascicles, and storiform arrays. Preliminary studies have shown that plain radiographs of the bone in PLPS presented with osteolytic lesions [10]. These findings are in accordance with our study, where the plain radiograph revealed osteolytic lesion at the right proximal femur with a minimally displaced pathological fracture at the intertrochanteric region.

Radiological investigations like MRI and CT are important to plan surgical resection in order to determine the size of the tumor and degree of invasion in neighboring tissues. [17]. However, it is challenging to differentiate PLPS from other types of liposarcoma and sarcoma, merely on the basis of these radiological examinations. The definite diagnosis of PLPS is made on the pathological examination. The presence of lipoblasts is necessary for diagnosis [14, 17]. In our case, the MRI showed the presence of a tumor at the proximal part of the right femur and its soft tissue invasion, especially at the posteromedial aspect. On histopathological analysis, variable size of pleomorphic adipocytes and lipoblasts was identified with vascular invasion. The differential diagnosis was metastasis carcinoma because the age of the patient was more than 40 years old and presented with painful osteolytic lesion and bone metastasis from soft tissue sarcoma is rare.

Surgery, especially wide resection that aims to achieve a negative surgical margin, is the main treatment for PLPS (Table 1) [17]. It is an important palliative treatment for PLPS patients with remote metastasis and local infiltration to alleviate the compressive manifestations [17]. Besides, the use of adjuvant chemotherapy and radiotherapy still remains controversial. Miura et al. reported that conventional chemotherapy was not useful for the sarcoma patient [18]. Some reports have suggested that the tumor volume and the local recurrence rate were reduced by radiotherapy, but on the other hand, the histopathology might be changed due to radiotherapy [17, 19].

On the contrary, a study shows poor outcomes in PLPS following surgery alone and recommends the use of neoadjuvant MAP chemotherapy [10]. In our case, surgical treatment involving wide resection that aimed to achieve a negative margin exhibited good oncological and functional outcomes in the patient. However, the effect of chemotherapy and radiotherapy in preventing postoperative recurrence is still unclear and requires further studies.

The data from previous studies show that metastasis from STS is considered as a hematogenous spreading and an end-stage of patients [20]. The distant metastasis rate in PLPS is 30–50% [5]. Bone metastasis from PLPS is not the most common site, reported in less than 10% of PLPS patients [21]. With regard to outcomes, PLPS is a highly aggressive tumor with a 5-year survival rate of only about 29% [17]. In our case, the tumor arose in the thigh of the patient at first and then metastasized to lungs, followed by recurrence and metastasis into the bone (femur). Radical resection of femoral metastasis gives good treatment results if a general health condition allows surgery [22].

## 4. Conclusion

PLPS is a rare high-grade sarcoma, more invasive tumor with high local recurrence and distant metastatic rate. However, the metastasis of PLPS to the bone is not the most common site. The definite diagnosis of PLPS is made based on histopathology. Surgical treatment involving wide resection that aims to achieve a negative margin is the best available option currently available, and we recommend treating bone metastasis from PLPS as the primary site of the tumor. The effect of chemotherapy and radiotherapy in preventing postoperative recurrence is still unclear and requires further studies.

## Figures and Tables

**Table 1:** Clinical, demographic features, type of treatments, results and outcomes of the present case, and reported cases of pleomorphic liposarcoma in the literature.

Study	Age (years)/sex	Location	Clinical data	Treatment	Result	Follow-up
Torok et al. [23]	34/M	Right femur	_	Wide resection, radiation, chemotherapy.	Resolution of the symptoms	Alive. 12 months follow-up.
Tiemeier et al. [10]	18/M	Metaphysis of the left tibia	Patient presented with a six-month history of pain and swelling in the left leg	Neoadjuvant MAP chemotherapy Surgical resection of the tumor	Complete resolution of the metastases following chemotherapy	12 months. Patient is well with no evidence of recurrence.
Rasalkar et al. [24]	13/M	Femur	_	Chemotherapy, surgical wide resection.	Resolution of symptoms	No recurrence at 13 months follow-up
Hamlat et al. [7]	45/F	Thoracic spine	Backache Paraplegia	Laminectomy T7-T8 and radiotherapy	Relieve from pain	19 months. Gradual deterioration of disease
Barra de Moraes et al. [15]	60/F	Lumbar spine	Lumbosciatica on the left side	Resection of L4, L3 to L5 arthrodesis. Radiotherapy and chemotherapy	At 18 months, neither pain nor recurrence	3 years. Lung metastasis
Morales-Codina et al. [14]	61/M	Lumbar spine	Bilateral lumbosciatica. Motor deficit in the lower limbs.	En bloc resection in L1, L2, and L3	Dehiscence and a deep wound infection. Inflammatory polymyopathy.	2 months. Local recurrence, hepatic metastasis, extensive thrombosis. Death
Present case	54/M	Right femur	Patient presented with a sudden right hip pain after he slipped.	En bloc proximal femur wide resection followed by cemented long-stem bipolar hemiarthroplasty. Radiotherapy and chemotherapy	All surgical margins were free from the tumor cell	6 months. Patient is well and could walk without gait aid. No evidence of recurrence or metastases.

## Data Availability

The data supporting this study's findings are available from the corresponding author upon reasonable request.
